# Study on Dynamic Mechanical Properties and Constitutive Model of Z-Shaped Steel Wire for Sealing Cable

**DOI:** 10.3390/ma19112180

**Published:** 2026-05-22

**Authors:** Ke-Yu Shen, Feng Fan, Xu-Dong Zhi, Rong Zhang

**Affiliations:** School of Civil Engineering, Harbin Institute of Technology, Harbin 150001, China; fanf@hit.edu.cn (F.F.); zhixudong@hit.edu.cn (X.-D.Z.); zhangrong_hit@163.com (R.Z.)

**Keywords:** Z-shaped steel wire, Hopkinson, Johnson–Cook constitutive model, model verification

## Abstract

This study investigates the flow stress behavior of Z-shaped steel wire used in cable sealing applications, over a temperature range of 20–500 °C and a strain rate range of 10^−4^ to 3000 s^−1^. The primary objective is to establish reliable constitutive data to support accurate numerical simulations in relevant engineering contexts. To this end, quasi-static tensile tests, high-temperature tensile tests, and high-strain-rate dynamic compression tests were conducted using a high–low temperature electronic universal testing machine and a split Hopkinson pressure bar system. The true stress–strain responses were obtained, and the corresponding mechanical properties were systematically analyzed. Experimental results show that at room temperature (20 °C) and within the low strain rate range (10^−4^–10^−1^ s^−1^), the flow stress is insensitive to strain rate variations. However, following yielding, the slope of the flow stress curve increases noticeably with accumulating strain, indicating deformation behavior governed predominantly by strain hardening. Under high-strain-rate conditions at room temperature (20 °C, 10^2^ to 10^3^ s^−1^), the yield stress increases with increasing strain rate, revealing a pronounced strain rate sensitivity. At elevated temperatures combined with a low strain rate (300–500 °C, 10^−3^ s^−1^), both the yield stress and the overall flow stress decrease markedly as the temperature rises, demonstrating significant thermal softening behavior. The microstructure and fracture of Z4 steel wire were observed by SEM to systematically investigate the effects of strain rate and temperature on its microstructural characteristics, thereby revealing the micro-mechanism of the material’s flow stress. Based on these experimental observations, a Johnson–Cook constitutive model was developed for the Z-shaped steel wire used in cable sealing applications. Validation results confirm that the model accurately captures the flow stress evolution of the material under coupled temperature and strain rate conditions.

## 1. Introduction

The dynamic response analysis of modern engineering structures under extreme loads, such as impact and explosion, has become the focus of research in the fields of civil engineering and protective engineering. Such dynamic events are usually accompanied by high strain rate, large deformation, and temperature effects, which cause the material to exhibit mechanical behavior that is completely different from that under quasi-static loading conditions. Accurately characterizing the dynamic constitutive relationship of metal materials under such complex stress states constitutes the key theoretical basis and a prerequisite for numerical simulation, essential for predicting the dynamic response of structures and evaluating their impact resistance and safety margin. Steel, as the most important load-bearing material in building and bridge structures, possesses dynamic mechanical properties, especially the strain rate sensitivity, temperature softening effect, and dynamic fracture mechanism of high-strength steel that have become core issues of current research [1,2].

In the field of dynamic constitutive model research, empirical and semi-empirical models are widely used because of their relatively simple form and easy calibration of parameters. Among them, the Johnson–Cook (JC) model is widely used to simulate the plastic flow behavior of various metal materials under impact load because of its consideration of strain hardening, strain rate strengthening, and temperature softening effects [3,4,5]. In the literature, many scholars have systematically calibrated and verified the JC model parameters of Q235 B, Q355 B construction steel, TB6, Ti-6Al-4V, and other titanium alloys by means of split Hopkinson pressure bar (SHPB) test and dynamic tensile test. In addition, other classical models, such as Bodner–Partom (BP) model [6,7], Zerilli–Armstrong (ZA) model [8,9], Khan–Huang (KH) model [10,11,12] and Steinberg–Guinan (SG) model [13,14,15], also describe the dynamic response of materials from the perspective of dislocation dynamics, physical mechanism or macroscopic phenomena, and each has its own applicable scenarios. However, most of the existing studies focus on conventional strength steels or aerospace alloys. For high-strength steel wires used for high-performance closed cables, there is still a lack of systematic and in-depth research on their dynamic constitutive relationship, micro-deformation mechanism, and failure criteria under extreme loads, which limits the accurate prediction of the impact resistance of closed cable members. Rypina et al. [16] present a novel methodology for optimizing Johnson–Cook constitutive model parameters through single abrasive grain micro-cutting experiments, providing more accurate material dynamic behavior for grinding process modeling. Tan et al. [17] enhanced the classical J–C model by incorporating a modification factor for the strain rate hardening coefficient, enabling a precise depiction of the flow characteristics of 7050 aluminum alloy across three varying high strain rate conditions. Bakhshan et al. [18] incorporated an ultrasonic amplitude- and frequency-dependent softening term into the classical J–C framework, proposing three modified J–C model variants, among which the multiplicative formulation demonstrated superior predictive accuracy under high strain conditions. Li et al. [19] modified the JC model by tensile test to describe the strain rate effect of DP1000 steel and verified its accuracy by the finite element method. Barik et al. [20] used the improved JC model to analyze the dynamic response of friction stir spot-welded AA5052-H32 thin plates, and the simulation results were in good agreement with the experimental results. Ghara et al. [21] established an indentation depth model based on JC flow strength and found that the influence of material flow strength on sandblasting performance was greater than that of hardness. Zhang et al. [22] characterized the dynamic mechanical behavior of titanium alloy TC4T based on the JC model and improved the model to apply to the stress analysis of aviation blades. Muniraj et al. [23] used the JC model to study the response and damage mechanism of honeycomb sandwich structures under impact. Liu et al. [24] systematically studied the dynamic mechanical properties of SWRH82B steel wire through experiments and numerical simulations. Most of the existing research focuses on conventional metal materials themselves, and there is a lack of systematic research on the dynamic constitutive, failure criteria, and microscopic mechanism of special cross-section members, such as high-strength steel wire Z-section members. The Johnson–Cook (JC) model is widely used to describe dynamic mechanical behavior due to its simple structure, clear physical meaning of parameters, and ease of calibration. It expresses flow stress as a product of strain hardening, strain-rate hardening, and thermal softening terms, which are decoupled from each other. Parameters can be independently determined via split Hopkinson bar and temperature-controlled tests, avoiding complex coupled identification. For the z-shaped steel wire material in this study, the JC model captures the main mechanical response with sufficient engineering accuracy. Moreover, many similar studies adopt the JC model, facilitating comparison with existing results. Therefore, the JC model is selected in this work.

The closed cable is made of high-carbon steel, and its unique Z-shaped steel wire bite structure leads to a complex internal contact state and stress distribution. At present, the research on cable members mostly focuses on static performance, relaxation effect, and cable clamp anti-slip performance, but the dynamic mechanical behavior under lateral impact load, especially the cross-scale correlation mechanism from material constitutive to component response, is not clear. Therefore, carrying out the dynamic mechanical properties test of Z-shaped steel wire, revealing its flow stress characteristics and fracture law at high strain rates, and then establishing or calibrating a high-precision dynamic constitutive model suitable for the material, is an indispensable cornerstone for systematically studying the lateral impact resistance of screw-shaped closed cables. The purpose of this paper is to systematically sort out the research context of the steel dynamic constitutive model and focus on the transient mechanical behavior of high-performance steel wire materials, to provide accurate material model support for the impact resistance design and numerical simulation of closed cable components.

## 2. Experimental Investigation

**Figure 1:**
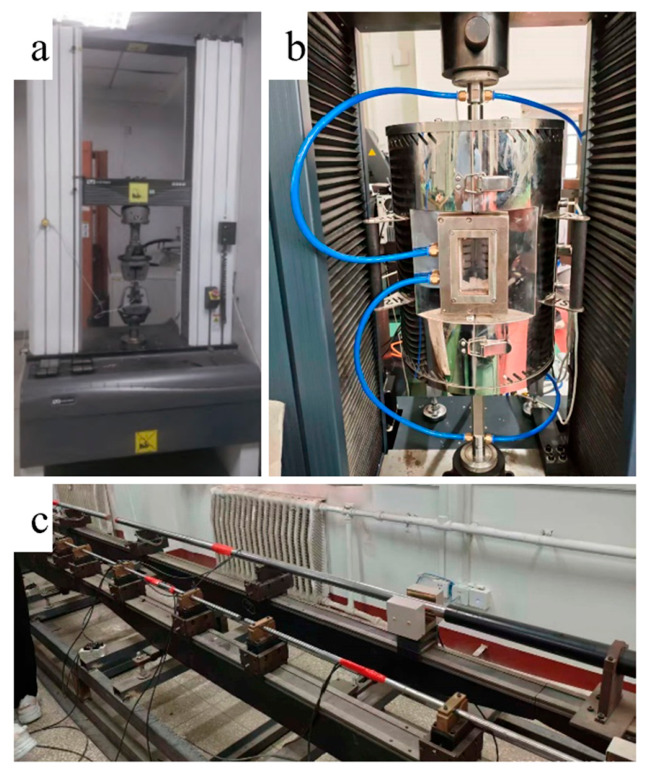
Experimental equipment. (**a**) Quasi-static tensile testing machine. (**b**) High-temperature stretching heating equipment. (**c**) Hopkinson pressure bar test equipment.

The quasi-static tensile and quasi-static high-temperature tensile tests were completed on the Instron3382 high and low-temperature electronic universal tensile test machine, (Instron Test Equipment Trading Co., Ltd., Shanghai, China) (Figure 1a). Among them, the quasi-static high-temperature tensile test needs to be combined with the use of a supporting high-temperature furnace device (Figure 1b). In addition, the dynamic compressive properties of the material under high strain rate conditions were tested by the Hopkinson pressure bar experimental system. The device is shown in Figure 1c. According to the specification ‘Metallic Materials Tensile Test Part 1: Room Temperature Test Method’ (GB/T 228.1-2021) and ‘Sampling Position and Sample Preparation for Mechanical Properties Test of Steel and Steel Products’ (GB/T 2975-2018), the tensile tests of three kinds of steel wires with different diameters at five different rates (1, 10, 50, 100, 200 mm/min) were carried out at room temperature of 20 °C. Each diameter wire was prepared in triplicate for a total of 45 tensile specimens.

**Figure 2:**
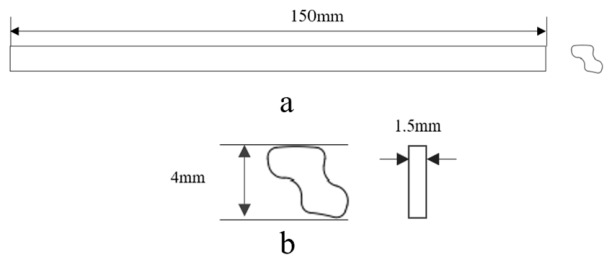
Specimens for mechanical experiments. (**a**) Quasi-static specimen for Z4C steel wire. (**b**) SHPB compression specimen for Z4C steel wire.

The nominal chemical elements of the Z4C steel wire are listed in Table 1. The test was carried out on an electronic universal testing machine, and the gauge distance of the extensometer for measuring strain was 20 mm. The Z-shaped steel wire specimen is cut from the straightened steel wire, with a gauge distance of 60 mm and a total length of 150 mm. Because the cross-section of Z-shaped steel wire is irregular and the material is hard and difficult to process, the standard tensile specimen shape in the specification is not applicable to Z-shaped steel wire. Therefore, the tensile test processing method for special cross-section wire in the specification is adopted. After the coil is straightened by physical means, it is directly intercepted as a tensile part, and the plate clamp is used for testing on the testing machine. The experimental specimen of the Hopkinson pressure bar is a Z-shaped cylinder with an equivalent diameter of 4 mm and a length of 1.5 mm. The experimental temperature is 20 °C, and the loading strain rate is 500–2200 s^−1^. Figure 2a shows the specimens with a Z-shaped section and a length of 150 mm that were used for the quasi-static elongation tests. Figure 2b shows the Z-shaped specimens with a diameter of 4 mm and a height of 1.5 mm produced for the SHPB test. The material grade of Z-shaped steel wire is 1670 MPa. For each group of tests, three specimens were used, and the results were calculated as the average of the three sets of data.

## 3. Results and Analysis

### 3.1. Quasi-Static Tensile Test at Room Temperature

**Figure 3:**
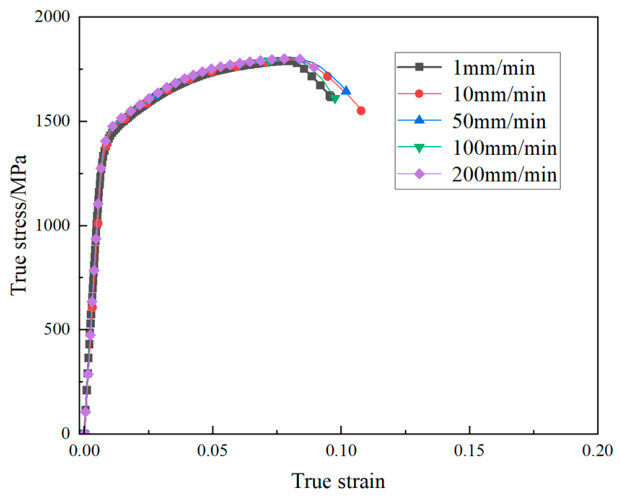
Stress–strain curve of Z-shaped steel wire.

In the quasi-static tensile test at room temperature, the specimen was tested by a high and low-temperature electronic universal tensile testing machine, and the true stress–strain curve of the material at a low strain rate was recorded by an automatic extensometer. The results are presented in Figure 3. The analysis indicates that under quasi-static loading conditions, the flow stress of the material does not vary significantly with increasing strain rate, demonstrating low strain rate sensitivity. After the material yields, as the strain continuously increases, the flow stress shows a significant upward trend, exhibiting a more significant strain hardening effect.

### 3.2. Hopkinson Pressure Bar Experiment

**Figure 4:**
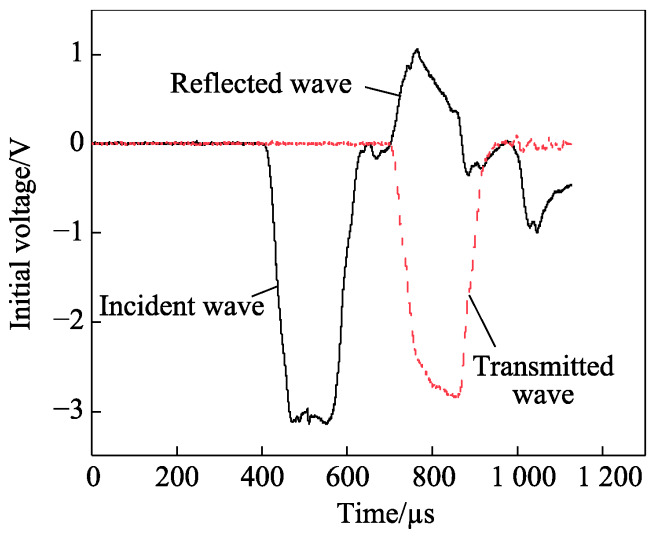
Typical waveforms of SHPB dynamic compression experiments recorded by an oscilloscope [17].

In order to study the strain rate effect of the material and its dynamic mechanical properties under high strain rate conditions, the impact compression experiment was carried out using the split hopkinson pressure bar (SHPB) (laboratory of Harbin Institute of Technology, Harbin, China). The experimental system mainly includes a gas gun, a bullet, an incident rod, a transmission rod, and a strain test device. By adjusting the gas gun pressure (0.1–0.7 MPa), the dynamic mechanical response data in the strain rate range of 500–2200 s^−1^ were obtained. The process of specimen deformation is based on the one-dimensional stress wave and uniform deformation hypothesis theory [25]. The typical waveform of an SHPB dynamic compression experiment recorded by an oscilloscope is shown in Figure 4.

**Figure 5:**
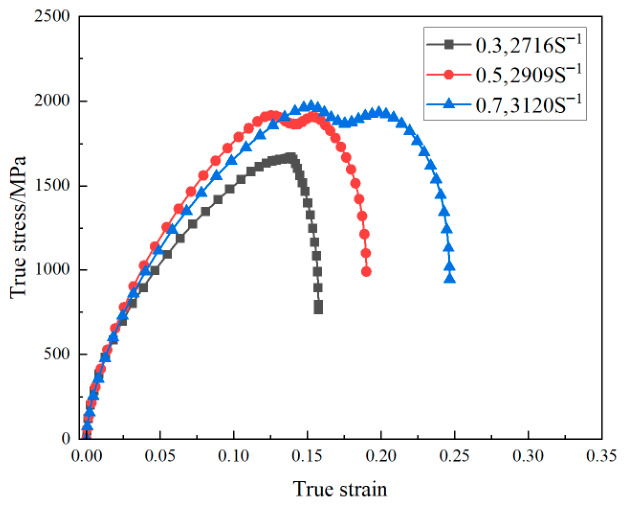
Stress–strain curve of Z-shaped steel wire at room temperature and high strain rate.

The waveform data in Figure 4 are processed to obtain the true stress–strain curves of the material at different strain rates, as shown in Figure 5. Figure 5 shows the stress–strain curves of Z-shaped steel wire at high strain rates of 0.3 MPa, 0.5 MPa, and 0.7 MPa. It can be seen from Figure 5 that with an increase in strain rate, the yield strength of the material increases due to the strain rate strengthening effect. At the same time, it is observed that the flow stress of the material after yield decreases and the failure strain increases, thus showing a larger strain range and enhanced toughness and ductility. For example, when the strain rate is 2716 s^−1^, the yield strength of the specimen is 1640 MPa; when the strain rate is about 2909 s^−1^, the yield strength of the material is about 1850 MPa, which is increased by 12.8%. When the strain rate is about 3120 s^−1^, the yield strength of the material is about 1905 MPa, which is increased by 2.97%. In addition, compared with low-yield-point steel, the strength of high-strength steel wire is slightly less sensitive to strain rate. At high strain rates, the stress–strain curve of the material shows a more obvious plastic platform, and the plastic deformation ability becomes better.

### 3.3. Quasi-Static High-Temperature Tensile Test

**Figure 6:**
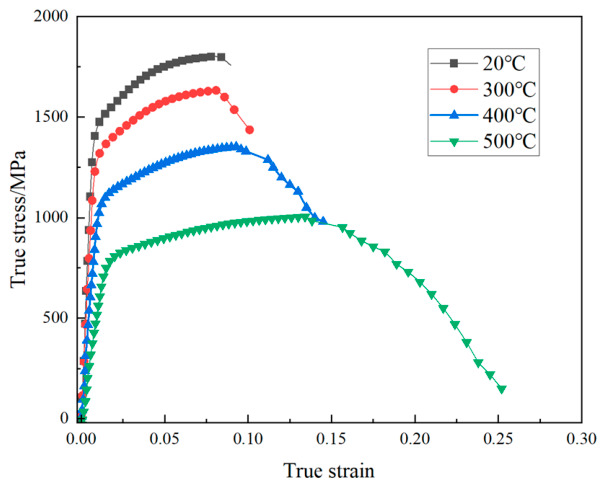
High-temperature tensile stress–strain curve of Z-shaped steel wire.

In order to explore the thermal softening behavior and high-temperature dynamic mechanical response of the material, high-temperature tensile experiments were carried out. The true stress–strain relationship of the material under different temperatures and quasi-static conditions was obtained by the software system of the universal tensile testing machine, as shown in Figure 6. It can be seen from Figure 6 that as the temperature increases, the stress–strain curve shows a significant overall downward shift, and the yield strength decreases significantly with an increase in temperature.

### 3.4. Fractography of Z-Shaped Steel Wire

**Figure 7:**
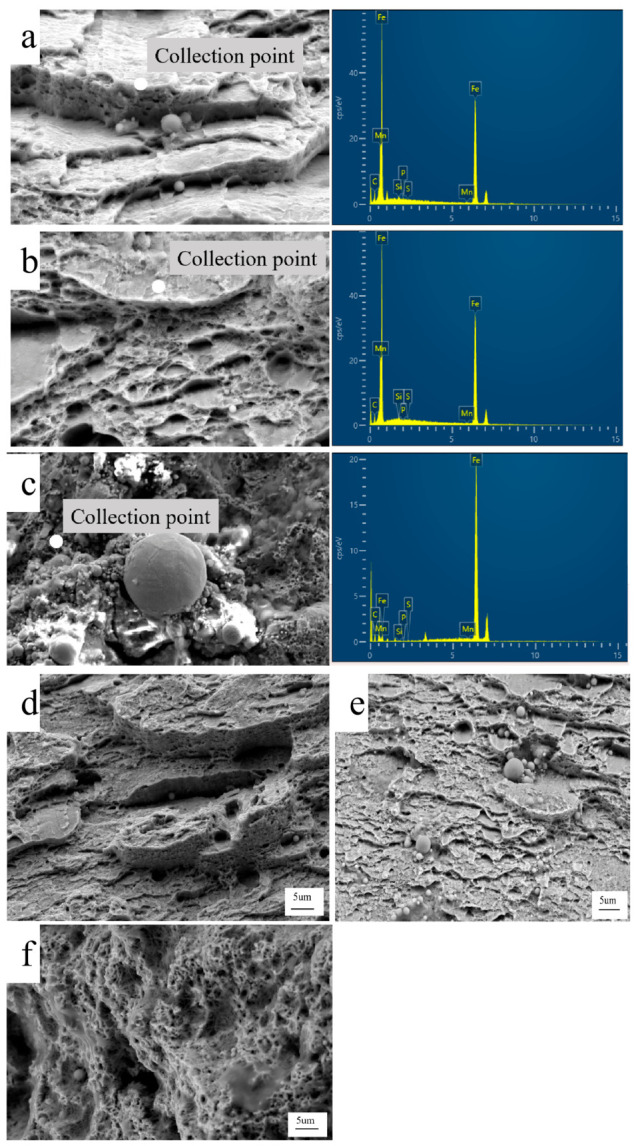
EDS mapping and SEM fractographs of Z-shaped steel wire tested at various strain rates and temperatures. EDS mapping. (**a**) T = 20 °C, 1 mm/min. (**b**) T = 20 °C, 100 mm/min. (**c**) T = 400 °C, 1 mm/min. SEM fractographs (**d**) T = 20 °C, 1 mm/min. (**e**) T = 20 °C, 100 mm/min. (**f**) T = 400 °C, 1 mm/min.

The stress–strain curves (Figure 3, Figure 5 and Figure 6) indicate that the deformation mechanism of the material depends on temperature and strain rate. Figure 7 shows the fracture morphology of the material after tensile deformation at different temperatures and strain rates, obtained by energy dispersion spectroscopy (EDS) elemental mapping and scanning electron microscopy (SEM). The results show that the content of Fe is the highest, while the content of C, Mn, and Si is lower than that in Table 1.

The fracture microstructure of Z-section steel wire is shown in Figure 7d at 20 °C and a loading speed of 1 mm/min. The results show that there are obvious cracks on the fracture surface, and the remaining areas are relatively smooth, showing typical brittle fracture characteristics. When the loading speed is increased to 100 mm/min, as shown in Figure 7e, the fracture also shows an obvious fracture surface and clearly visible cracks, still showing brittle fracture characteristics. This phenomenon is highly consistent with the results under the condition of 1 mm/min, indicating that the loading speed has little effect on the mechanical properties of the material under quasi-static loading conditions, which is consistent with the experimental results in Figure 3. Figure 7f is the fracture microstructure at 400 °C and a 1 mm/min loading rate. It can be seen from the figure that the fracture surface is no longer smooth, but presents a honeycomb micro-pit structure, and the honeycomb surface is distributed with spherical particles formed by the oxidation of iron crystals.

Inside the material crystals, there is resistance that hinders dislocation movement, so an external force is needed to drive dislocation movement. When the atom moves from one equilibrium position to another, sufficient energy must be obtained to overcome the energy barrier. The height of these barriers and the lattice thermal vibration energy will affect the temperature distribution and strain transmission path. Increasing the test temperature will increase the thermal vibration amplitude of the atoms in the steel wire, thereby reducing the resistance to dislocation motion, helping the atoms to cross the barrier, making the atoms more active and the dislocation motion more likely to occur. Macroscopically, the increase in temperature leads to a decrease in ultimate strength, that is, the temperature softening effect; at the same time, the section is transformed from the ductile–brittle mixed region to the completely ductile region, which is manifested as crack-free ductile failure, and the elongation is significantly increased. The strain rate effect is opposite to temperature softening. It takes time for atoms to overcome the barrier. The time window at high strain rates is extremely short, such that dislocation motion is rendered less likely to occur, and the opportunity to absorb energy is reduced. Therefore, it is more difficult for materials to overcome dislocation motion, and the ultimate strength is improved, that is, the strain rate hardening effect. With an increase in strain rate, the fracture surface transforms from the ductile–brittle mixed region to the completely brittle region, showing brittle fracture.

## 4. Constitutive Model and Parameter Calibration

### 4.1. Constitutive Model of Material

In order to obtain the necessary mechanical parameters of the material in the finite element simulation, based on the material mechanical performance experiment, the Johnson–Cook constitutive model is used to describe the dynamic mechanical behavior of the material, and the specific expression is shown in Formula (1):

(1) $$\sigma =\left(A+B\varepsilon^{n}\right)\left(1+Clin\dot{\varepsilon }*\right)\left(1-{T*}^{m}\right)$$

In the formula: $\dot{\varepsilon }*$ is the dimensionless strain rate, $\dot{\varepsilon }*=\dot{\varepsilon }/{\dot{\varepsilon }}_{0}$, where $\dot{\varepsilon }$ is the current strain rate and ${\dot{\varepsilon }}_{0}$ is the reference strain rate. T* is the dimensionless temperature, $T*=\left(T-T_{r}\right)/\left(T_{m}-T_{r}\right)$, where T, $T_{m}$ and $T_{r}$ are respectively the current temperature, melting point temperature, and reference temperature of the material; A, B, *n*, C, and m are five coefficients to be determined in the constitutive model. A is the initial yield stress of the material, that is, the yield limit; B is the hardening modulus, representing the material’s resistance to further deformation as it undergoes plastic deformation; *n* is the hardening index, which determines the shape of the hardening curve; C is the strain rate sensitivity coefficient. The larger the C value, the more significant the increase in material strength at high strain rates; m is the thermal softening index, which determines the rate at which the material’s strength decreases with increasing temperature. The essence of the Johnson–Cook model lies in its decoupling of strain hardening, strain rate hardening, and thermal softening effects in the form of a product, which makes it simple in form and easy to implement in finite element programs. The three parentheses on the right side of the above equation correspond to these three effects, respectively. The first term: strain hardening term $\left(A+B\varepsilon^{n}\right)$; the second term: strain rate sensitivity term $\left(1+Clin\dot{\varepsilon }*\right)$; the third term: thermal softening term $\left(1-{T*}^{m}\right)$.

### 4.2. Constitutive Model Fitting

When fitting the first term of the J–C constitutive model, the quasi-static tensile experimental data at room temperature at the reference strain rate are selected: the temperature is 20 °C, and the strain rate is 0.001 s^−1^. At this time, the strain rate term and the temperature term are both 1, and Equation (1) can be simplified as Equation (2)

(2) $$\sigma_{eq}=A+B\varepsilon_{eq}^{n}$$

After a simple transformation, it can be obtained that:

(3) $$ln\left(\sigma_{eq}-A\right)=lnB+nln\varepsilon_{eq}$$

**Figure 8:**
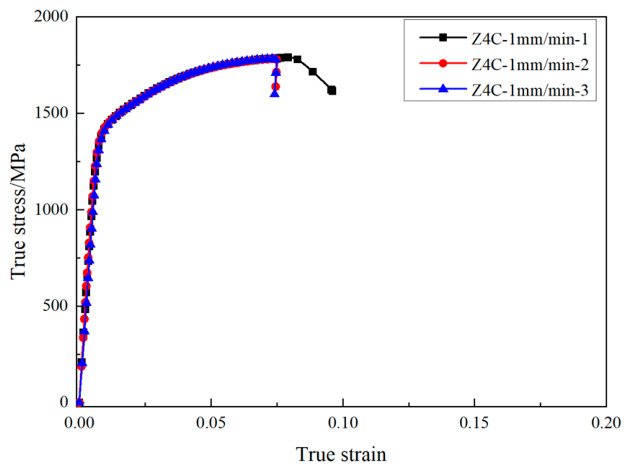
True stress–strain curve of Z-shaped steel wire at room temperature and low strain rate.

Note that only the logarithm of a positive number can be taken, so only the data after the material yields are fitted. A is the yield stress of the specimen in the single tensile test. For the Z4C steel wire, the corresponding data are shown in Figure 8 and Table 2. Thus, A is 1395.5 MPa.

According to the data in Table 2, the yield strength of Z-shaped steel wire σ is 1395.5 MPa, the ultimate tensile strength σ_u_ is 1785.3 MPa, and the elastic modulus *E* is 204,842 MPa.

Because there is no obvious yield platform during the tensile process of the specimen, the yield strength is determined by the corresponding stress when the residual plastic strain is 0.2%. Therefore, the equivalent plastic strain:

(4) $$\varepsilon_{eq}=\varepsilon -\sigma /E-0.2\%$$

**Figure 9:**
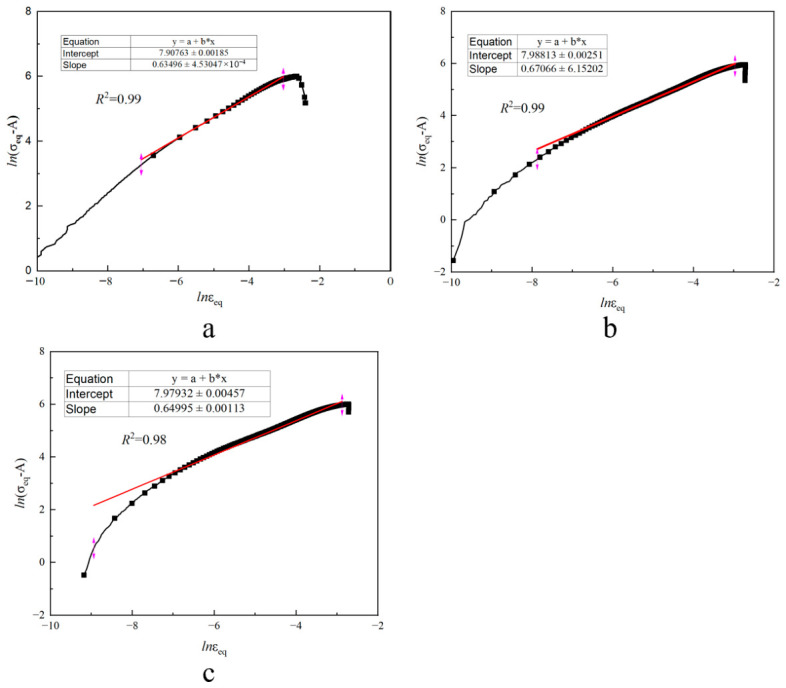
Linear fitting of the logarithmic stress–strain curve of Z-shaped steel wire at room temperature. (**a**) 1 mm/min^−1^. (**b**) 1 mm/min^−2^. (**c**) 1 mm/min^−3^.

Based on Formula (4), the stress and strain are logarithmically transformed. The logarithmic strain versus logarithmic stress curve is then plotted and fitted, as shown in Figure 9.

The intercept ln *B* and slope *n* of each curve are counted in Table 3.

In summary, the Z-shaped steel wire J–C model: *A* = 1.3955 GPa, *B* = *e*^7.96^ = 2864 Pa, *n* = 0.67.

For the Hopkinson pressure bar impact test at the reference temperature, Formula (1) can be written as:

(5) $$\sigma_{eq}=\left(A+B\varepsilon_{eq}^{n}\right)\left(1+Cln\left({\dot{\varepsilon }}_{eq}/{\dot{\varepsilon }}_{0}\right)\right)$$

At the yield point, $\varepsilon_{eq}=0$, the above equation can be rewritten as:

(6) $$\sigma_{eq}=A\left(1+Cln\left({\dot{\varepsilon }}_{eq}/{\dot{\varepsilon }}_{0}\right)\right)$$

(7) $$\sigma_{eq}/A=1+Cln\left({\dot{\varepsilon }}_{eq}/{\dot{\varepsilon }}_{0}\right)$$

**Figure 10:**
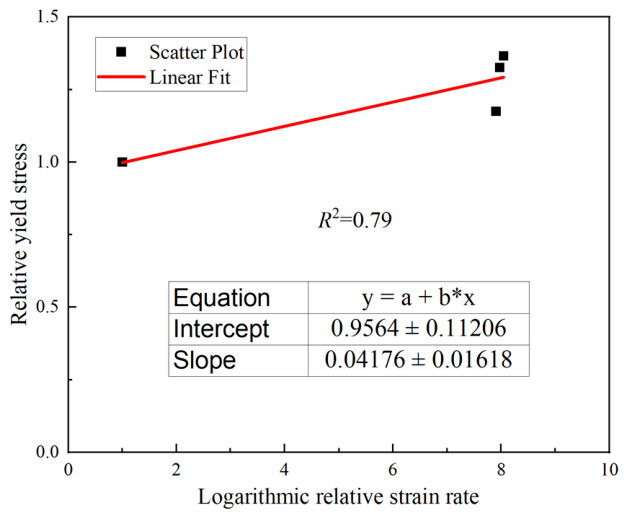
Parameter C fitting.

According to the experimental data, the image of the relationship is made, as shown in Figure 10. Linear fitting curve, the slope is the parameter C. It can be seen from Figure 10 that the J–C model parameter of Z-shaped steel wire is C = 0.04176.

In the quasi-static tensile test at high temperature, the strain rate of the specimen is the same as that of the quasi-static tensile test at room temperature, that is, the reference strain rate. Under the reference strain rate, the basic form of the Johnson–Cook constitutive model is rewritten to obtain the relationship between yield stress and temperature, as shown in Formula (8):

(8) $$ln\left(1-\sigma_{eq}/A\right)=mlnT^{*}$$

**Figure 11:**
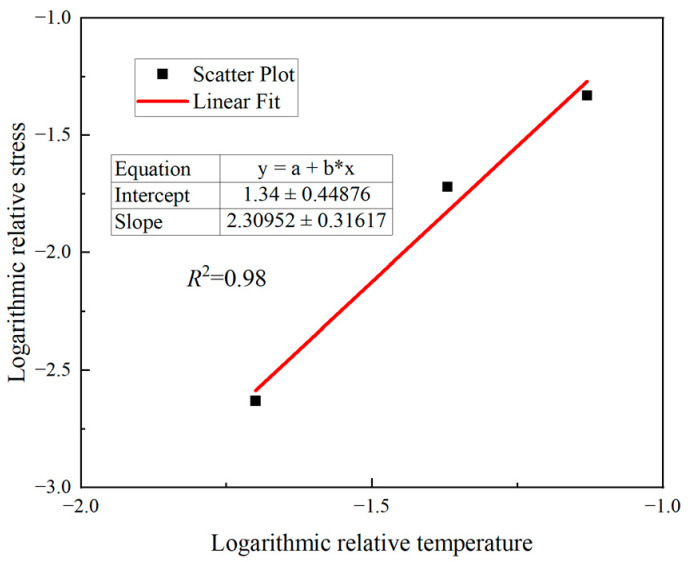
Parameter m fitting.

According to the yield strength of the material at different temperatures analyzed by the test results, the relationship between $ln\left(1-\sigma_{eq}/A\right)$ and $lnT^{*}$ is fitted, and a straight line is fitted. The slope of the straight line is the temperature-related parameter m. The scatter plot of the parameter relationship and the fitting results are shown in Figure 11. It can be concluded that *m* = 2.31.

Based on the above results, the final form of the JC model is:

(9) $$\sigma =\left(1.3955e^{9}+2.864e^{3}\varepsilon^{0.67}\right)\left(1+0.04176ln\dot{\varepsilon }*\right)\left(1-{T*}^{2.31}\right)$$

### 4.3. Fitting Results

**Figure 12:**
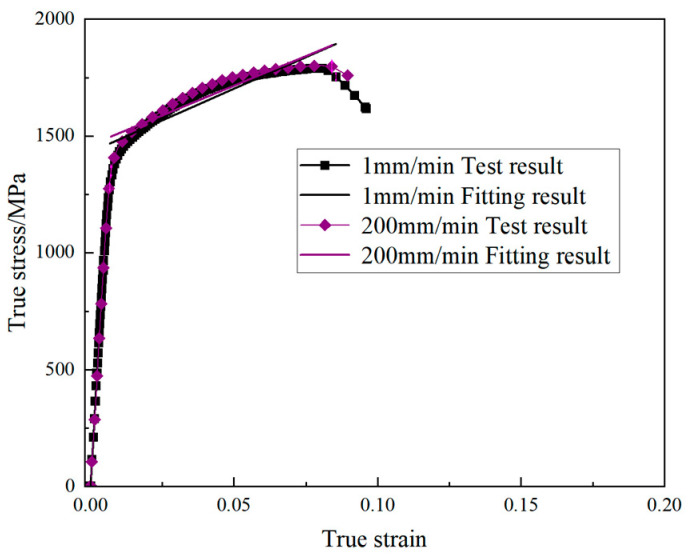
Comparison of experimental data and fitting results at room temperature and low strain rate.

**Figure 13:**
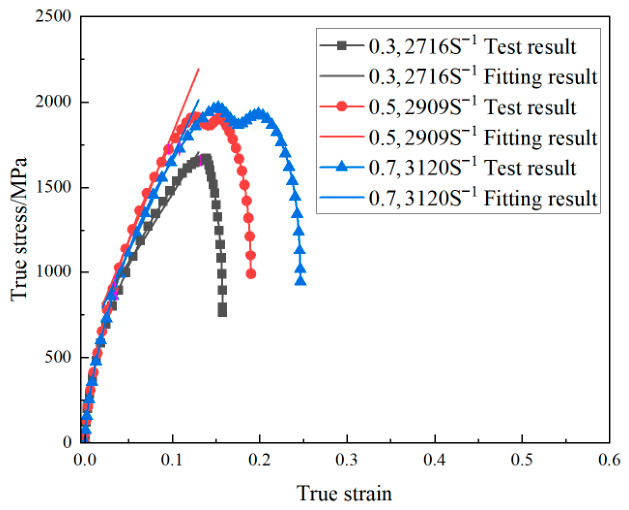
Comparison of experimental data and fitting results at room temperature and high strain rate.

**Figure 14:**
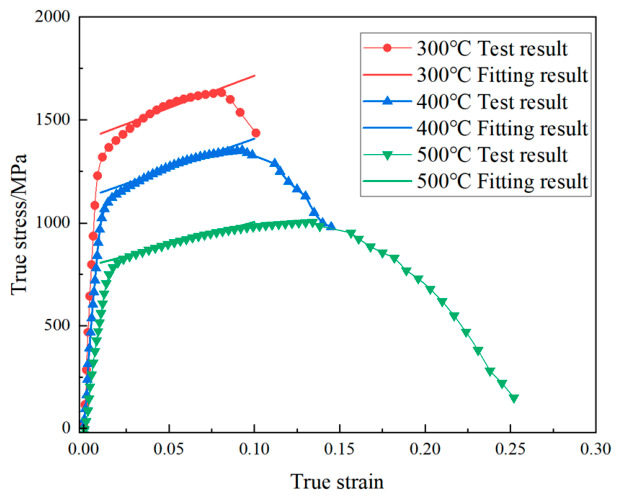
Comparison of experimental data and fitting results at different temperatures at the tensile rate of 1 mm/min.

The Johnson–Cook constitutive model aims to characterize the true stress–strain response of the material under different deformation conditions. By substituting the strain data into the model equation, the corresponding stress–strain curve can be fitted, and then the accuracy of the model is evaluated by comparing the fitting curve with the experimental data. Figure 12 shows the comparison between the quasi-static tensile test data at room temperature and the fitting results of the Johnson–Cook model: for 1 mm/min, *R*^2^ = 0.92; for 200 mm/min, *R*^2^ = 0.98. The two are basically coincident on the whole, indicating that the model has good description ability under this condition. Figure 13 further compares the experimental data and model fitting results at different high strain rates at room temperature. It can be seen that the true stress–strain curve corresponding to each strain rate is basically consistent with the fitting curve in the trend of change, for 0.3, *R*^2^ = 0.96; for 0.5, *R*^2^ = 0.95; for 0.3, *R*^2^ = 0.97. Figure 14 compares the experimental data and model fitting results at different temperatures under quasi-static conditions: for 300 °C, *R*^2^ = 0.91; for 400 °C, *R*^2^ = 0.93; for 500 °C, *R*^2^ = 0.99. Due to the stress drop phenomenon of the material in the high-temperature tensile experiment, the fitting results show a certain deviation with an increase in strain, but the overall change trend is still well reflected.

## 5. SHPB Simulation Study on Dynamic Mechanical Properties of Z-Shaped Steel Wire

In order to test the reliability and accuracy of the established J–C constitutive model of Z-shaped steel wire in the high strain rate domain under the condition of large strain rate, the parameters of the modified J–C constitutive model obtained above are imported into the finite element software to simulate the SHPB compression process of Z-shaped steel wire at different strain rates, and the simulation results are compared with the experimental data.

### 5.1. Finite Element Model

**Figure 15:**
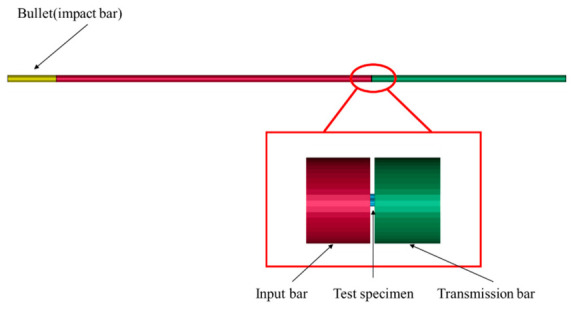
Finite element model of the SHPB experiment.

According to the experimental conditions, a three-dimensional finite element model is established as shown in Figure 15. The SHPB experimental components include bullets, incident rods, specimens, and transmission rods. The diameter of the bullet is 14.5 mm, and the length is 200 mm. The diameter of the incident rod is 14.5 mm, and the length is 1300 mm. The equivalent diameter of the specimen is 4 mm, and the length is 1.5 mm. The diameter of the projection rod is 14.5 mm, and the length is 800 mm. Since the bullets, incident rods, and transmission rods of the SHPB device are all made of the same material, the elastic constitutive model in the linear module can be used to describe the rod. The specific material parameters are shown in Table 4. The specific parameters of Z4C steel wire can be calculated by the relationship equation shown in Formula (9).

According to the experimental conditions of the split Hopkinson pressure bar (SHPB) and the requirements of numerical simulation modeling, the contact type should be set to surface-to-surface automatic contact to more accurately reflect the contact state in the actual SHPB experiment. At the same time, the friction effect between the contact interfaces of each bar can be ignored. Because the strain rate parameter is difficult to directly apply in finite element software, the loading speed measured by the experiment can be used as an approximate replacement. In the simulation model, the loading is realized by giving the initial speed of the bullet along the direction of the incident rod. The value is consistent with the experimental acquisition speed value. According to the experimental record, when the strain rate is 2716 s^−1^, 2909 s^−1^, and 3120 s^−1^, the corresponding speeds of the bullet are 4.13 m/s, 6.37 m/s, and 9.25 m/s, respectively. In order to ensure that the loading conditions of the sample are consistent with the actual SHPB experiment, it is necessary to ensure that the incident wave and the reflected wave do not superimpose during a complete stress wave propagation process, that is, the sample needs to go through three stages of incident, reflection, and transmission in turn. After calculation and analysis, the simulation time range is set to 0–400 μs, which can meet the integrity requirements of the above propagation process.

### 5.2. The Simulation Results Are Compared with the Experimental Results

**Figure 16:**
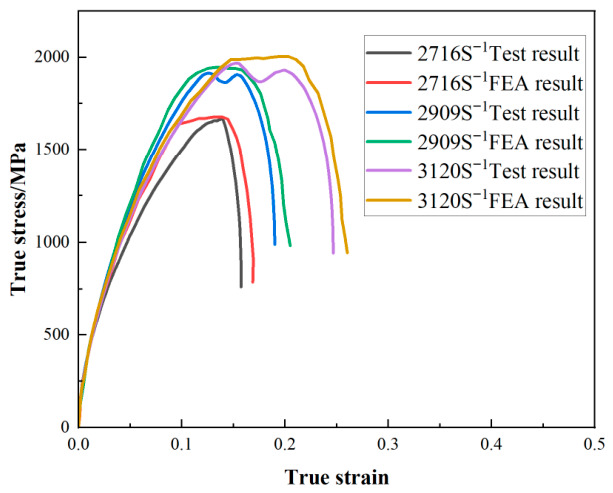
Comparison of SHPB experimental results with SHPB numerical simulation results.

In order to verify the rationality and reliability of the modified Johnson–Cook constitutive model, the stress–strain curve obtained by numerical simulation based on the model is compared with the experimental data. The results are shown in Figure 16. In the stress–strain curve, there are clear elastic-plastic stages and failure stages under different strain rates, and the yield stress amplitude increases with an increase in strain rate. The experimental data and numerical simulation data are basically the same.

Based on the comparison of the stress–strain curves between the simulation and the experiment, it can be seen that the numerical simulation of the SHPB compression experiment of Z4 steel wire using the modified Johnson–Cook constitutive model has high accuracy and reliability and can truly reflect the mechanical response characteristics of this type of steel wire in the dynamic compression process.

## 6. Conclusions

In this work, the mechanical response behavior of Z-shaped steel wire used for cable sealing was systematically investigated through a series of well-designed mechanical experiments. These included quasi-static tensile tests at room temperature (strain rates of 10^−4^–10^−1^ s^−1^), quasi-static high-temperature tensile tests (300–500 °C), and dynamic compression tests using a split Hopkinson pressure bar system (strain rates of 500–2200 s^−1^). The experimental data were processed and analyzed to obtain true stress–strain curves. The main conclusions are as follows:

(1) At room temperature, the Z-shaped steel wire exhibits a pronounced strain hardening effect under both low and high strain rate conditions. Following yielding, the flow stress increases progressively with strain, and the tensile strength, reaching up to 1875 MPa, is substantially higher than the yield strength.
(2) Comparison of quasi-static and high-strain-rate experimental data reveals that the flow stress of the Z-shaped steel wire is highly sensitive to strain rate variations, demonstrating a significant strain rate strengthening effect.
(3) The material also shows strong thermal softening behavior. As temperature increases, the flow stress decreases notably, while the strain of failure (unloading strain) increases accordingly.
(4) Based on the experimental results from room-temperature quasi-static tension, room-temperature high-strain-rate tension, and high-temperature tension, the parameters of the Johnson–Cook constitutive model for the Z-shaped steel wire were calibrated as follows: *A* = 1395.5 MPa, *B* = 3.041 MPa, *n* = 0.67, *C* = 0.04176, and *m* = 2.31. The model accurately captures the evolution of flow stress from yield to the onset of necking, thereby providing a reliable foundation for numerical and finite element simulations of Z-shaped steel wire under various loading conditions.
(5) Furthermore, the calibrated Johnson–Cook model effectively describes the dynamic compressive behavior of the Z-shaped steel wire as characterized by SHPB tests, confirming its reliability for predicting material response under high-strain-rate deformation.

## Figures and Tables

**Table 1:** Chemical elements of Z4C steel wire.

Element	C	Mn	Si	P	S	Fe
Contents (wt.%)	0.84	0.71	0.23	0.02	0.01	Bal.

**Table 2:** Mechanical properties of Z4C steel wire.

Specimen Number	1 mm/min^−1^	1 mm/min^−2^	1 mm/min^−3^	Mean Value
Yield strength	1388.5	1396.9	1401.0	1395.5
Elastic modulus	208,690	209,062	196,773	204,841
Ultimate strength	1789.9	1782.8	1783.3	1785.3

**Table 3:** Z-shaped steel wire curve index.

	1 mm/min^−1^	1 mm/min^−2^	1 mm/min^−3^	Mean Value
ln *B*	7.91	7.99	7.97	7.96
*n*	0.63	0.67	0.71	0.67

**Table 4:** Material properties of SHPB experimental components.

Component Material	Density/(kg/m^3^)	Elastic Modulus/GPa	Poisson Ratio
High-strength steel	7830	210	0.3

## Data Availability

The original contributions presented in this study are included in the article. Further inquiries can be directed to the corresponding author.
